# Association of polygenic risk scores and hair cortisol with mental health trajectories during COVID lockdown

**DOI:** 10.1038/s41398-022-02165-9

**Published:** 2022-09-21

**Authors:** Kira F. Ahrens, Rebecca J. Neumann, Nina M. von Werthern, Thorsten M. Kranz, Bianca Kollmann, Björn Mattes, Lara M. C. Puhlmann, Danuta Weichert, Beat Lutz, Ulrike Basten, Christian J. Fiebach, Michèle Wessa, Raffael Kalisch, Klaus Lieb, Andreas G. Chiocchetti, Oliver Tüscher, Andreas Reif, Michael M. Plichta

**Affiliations:** 1 Goethe University Frankfurt, University Hospital, Department of Psychiatry, Psychosomatic Medicine and Psychotherapy, Frankfurt, Germany; 2 Department of Psychiatry and Psychotherapy, University Medical Center Mainz, Mainz, Germany; 3 Leibniz Institute for Resilience Research (LIR), Mainz, Germany; 4 Institute of Psychology, Technical University of Darmstadt, Darmstadt, Germany; 5 Institute of Physiological Chemistry, University Medical Center Mainz, Mainz, Germany; 6 Department of Psychology, Goethe University Frankfurt, Frankfurt am Main, Germany; 7 Brain Imaging Center, Goethe University, Frankfurt, Germany; 8 Department of Clinical Psychology and Neuropsychology, Institute for Psychology, Johannes Gutenberg University Mainz, Mainz, Germany; 9 Neuroimaging Center (NIC), Focus Program Translational Neuroscience (FTN), Johannes Gutenberg University Medical Center Mainz, Mainz, Germany; 10 Department of Child and Adolescent Psychiatry, Psychosomatics and Psychotherapy, Goethe University Frankfurt, Frankfurt, Germany

**Keywords:** Predictive markers, Human behaviour, Clinical genetics

## Abstract

The COVID-19 pandemic is a global stressor with inter-individually differing influences on mental health trajectories. Polygenic Risk Scores (PRSs) for psychiatric phenotypes are associated with individual mental health predispositions. Elevated hair cortisol concentrations (HCC) and high PRSs are related to negative mental health outcomes. We analyzed whether PRSs and HCC are related to different mental health trajectories during the first COVID lockdown in Germany. Among 523 participants selected from the longitudinal resilience assessment study (LORA), we previously reported three subgroups (acute dysfunction, delayed dysfunction, resilient) based on weekly mental health (GHQ-28) assessment during COVID lockdown. DNA from blood was collected at the baseline of the original LORA study (*n* = 364) and used to calculate the PRSs of 12 different psychopathological phenotypes. An explorative bifactor model with Schmid-Leiman transformation was calculated to extract a general genetic factor for psychiatric disorders. Hair samples were collected quarterly prior to the pandemic for determining HCC (*n* = 192). Bivariate logistic regressions were performed to test the associations of HCC and the PRS factors with the reported trajectories. The bifactor model revealed 1 general factor and 4 sub-factors. Results indicate a significant association between increased values on the general risk factor and the allocation to the acute dysfunction class. The same was found for elevated HCC and the exploratorily tested sub-factor “childhood-onset neurodevelopmental disorders”. Genetic risk and long-term cortisol secretion as a potential indicator of stress, indicated by PRSs and HCC, respectively, predicted different mental health trajectories. Results indicate a potential for future studies on risk prediction.

## Introduction

The etiology of stress-related disorders is multifactorial and likely depends on both genetic and environmental factors [1, 2]. Few studies so far have examined the combined effect of different polygenic risk scores (PRSs) and hair cortisol concentration (HCC) [3, 4], a highly heritable indicator of long-term cortisol secretion and a potential indicator of environmental stress, to identify people at-risk for developing mental disorders. There is clear evidence that adverse experiences and stress can induce stress-related disorders [5]. However, individuals show differential mental health trajectories after adverse events [6]. Currently, it is unclear whether these different trajectories can be predicted by genetic predisposition and/ or long-term cortisol secretion and stressability. In an earlier investigation, we identified three subgroups with differing mental health trajectories during the first COVID lockdown in Germany and two groups emerged as particularly at increased risk of developing a mental disorder, while exposed to the same amount of COVID-19 specific stressors [7, 8]. To understand the potential (patho-)mechanisms defining these groups, we here investigated the association between these subgroups with both HCC as well as PRSs for phenotypes of mental disorders and traits.

Stress, an established risk factor for mental disorders [5] is known to activate the hypothalamus-pituitary-adrenal (HPA) axis. HPA axis activation is highly heritable and can be measured by its end product, the steroid hormone cortisol [4]. As cortisol is incorporated into growing hair, long-term cortisol secretion can retrospectively be measured by HCC [9] and HCC may be a promising indicator of environmental stress and stressability [9]. While previous studies have shown increased HCC in individuals with different objectively observable stressors [10–14], current meta-analyses have shown inconsistent results regarding associations of HCC with stress-related disorders [9, 15, 16]. However, only very few studies have examined the predictive value of actually past cortisol levels for future stress reactions in prospective longitudinal designs (exception see [17]). In most so-called “prospective” studies hair samples were collected immediately after the traumatic event. Since 3 cm hair samples express cumulative cortisol secretion over three months preceeding the event, they are referred to as pretraumatic HCC levels [18, 19]. Regarding this particular time of sampling, it is unknown if the HCC values confound with acute cortisol secretion during the traumatic event. The present understanding of HCC as a potential predictor for stress vulnerability is therefore still limited.

In addition to HCC, the genetic predisposition of individuals for developing mental disorders is another important risk factor. Genome-wide association studies (GWAS) provide strong evidence of multiple independent loci contributing additively to the etiology of different psychiatric disorders [20]. To determine this genetic risk at the individual level, polygenic risk score (PRS) can be calculated [21], defining single values to quantify an individual’s additive genetic trait predisposition. Schultebraucks et al. [22] used 21 PRSs and supervised machine learning and predicted mental health trajectories following a major life event on the basis of multiple PRSs. Moreover, among others, the Brainstorm Consortium [23], as well as the Cross-Disorder Group of the Psychiatric Genomic Consortium [24, 25] pointed to pleiotropic mechanisms among psychiatric disorders. For instance, Lee et al. provided strong empirical evidence for the existence of pleiotropic mechanisms by combining eight different neuropsychiatric disorders using exploratory factor analysis into three factors that explained 51% of the variance [26]. These pleiotropic mechanisms are not an entirely new concept. Additionally, a general factor for psychopathology has already been established, following the concept of the general factor in intelligence (g factor) [27, 28]. Various studies demonstrated that different mental disorders directly loaded on an orthogonal general bifactor [27–29]. A more recent example is the study by Grotzinger et al. [29]. In this study the possibility of describing 11 major psychiatric disorders by a bifactor model with four domain-specific factors and one p-factor was demonstrated [29]. However, many important issues, such as adequate integration of different genetic risk factors or the combination of genetic risk for psychiatric disorders with other types of biomarkers, have remained unexplored [30]. The present study aimed at tackling these issues by integrating HCC as an indicator of long-term cortisol secretion and a potential outcome of environmental stress as well as the PRSs as indicators of genetic risk for psychiatric disorders, while at the same time considering the interaction of HCC and PRSs. We included this interaction because identifying individuals in whom environmental conditions such as stress increase genetic influence would be very helpful in preventing psychiatric disorders. Moreover, it has recently received attention by a study from Bolhuis et al., who examined the moderating effect of HCC on the association of the genetic risk of schizophrenia with pre-adolescent brain structure [3]. In this study the interaction effect did not surpass the multiple testing threshold [3] and therefore remained inconclusive. In addition, Rietschel and colleagues showed no association between HCC and the PRSs for MDD or the PRS for neuroticism [4]. We reasoned that the investigation of this interaction would therefore be beneficial as an additional exploratory analysis.

This study builds on data from the LOngitudinal Resilience Assessment (LORA)-study [31]. To quantify stress vulnerability, we referred to three groups of participants with different mental health trajectories during the first 8 weeks of lockdown of the COVID-19 pandemic that were identified in previous work: [7, 8] the largest class with 82.6% is the “resilient” class, which maintained a level of low mental distress due to the major stressor. The smallest group, with 8.4%, showed significant deterioration of mental health after week 3 and is therefore called “delayed dysfunction”. The last class entails 9.0% of the participants and initially reacted with significant mental health deterioration but an improvement beyond the baseline level from week 5. In this study, we will call the class of participants “acute dysfunction”, as it reacts immediately with a stress response to the pandemic and associated socio-political restrictions. Noteworthy, the two groups that reacted with a significant deterioration of mental health during the pandemic also had significantly worse mental health values prior to the pandemic compared to the resilient class [7]. Accordingly, both groups (“acute dysfunction” and “delayed dysfunction”) are considered to represent vulnerable individuals that should have been identified in time and supported through appropriate interventions.

To account for the high genetic correlation of different psychiatric disorders mentioned above, we decided to use PRSs for different mental disorders and an associated personality trait: attention deficit hyperactivity disorder (ADHD) [32], alcohol dependence (ALC) [33], anorexia nervosa (ANO) [34], anxiety disorder (ANX) [35], autism spectrum disorder (AUT) [36], bipolar disorder (BPD) [37], major depressive disorder (MDD) [38], obsessive-compulsive disorder (OCD) [39], opioid dependence (OPI) [40], posttraumatic stress disorder (PTSD) [41], schizophrenia (SCZ) [42], and neuroticism (NEU) [43]. NEU was added to this comprehensive set of the most common psychiatric disorders, because it is a risk factor for a variety of psychiatric disorders, e.g., depression and schizophrenia [43]. To capture the pleiotropic mechanisms among psychiatric disorders, we followed the bifactor model and additionally computed the risk for the general p factor based on the 12 different PRSs.

This current study thus seeks to investigate the utility of prospectively sampled biological parameters to identify individuals belonging to a group of participants with deteriorated mental health after the outbreak of the COVID-19 pandemic in Germany.

According to earlier evidence linking HCC to stress-related disorders and the development of associated symptoms, we hypothesize (1) that elevated pre-pandemic HCC are associated with their allocation to the “acute dysfunction” and the “delayed dysfunction” classes during the pandemic. In addition, we hypothesize (2) that subjects with a general increased genetic risk for developing a psychiatric disorder will also be more likely to have deteriorating mental health in response to the COVID lockdown. Furthermore, we exploratorily test (3) the impact of disorder-specific PRSs and (4) whether there is an interaction effect of long-term cortisol secretion and genetic risk, i.e. that the combination of high cortisol levels in hair, physiological differences and high genetic risk for developing a mental illness are more likely to occur in vulnerable individuals compared to resilient individuals.

## Materials and methods

### Study design

The present study is an extension of the **LO**ngitudinal **R**esilience **A**ssessment (LORA)-study, which is ongoing since 2017. For a more detailed description of the LORA study, see the Supplemental Material and Chmitorz et al. [31].

**Fig. 1:**
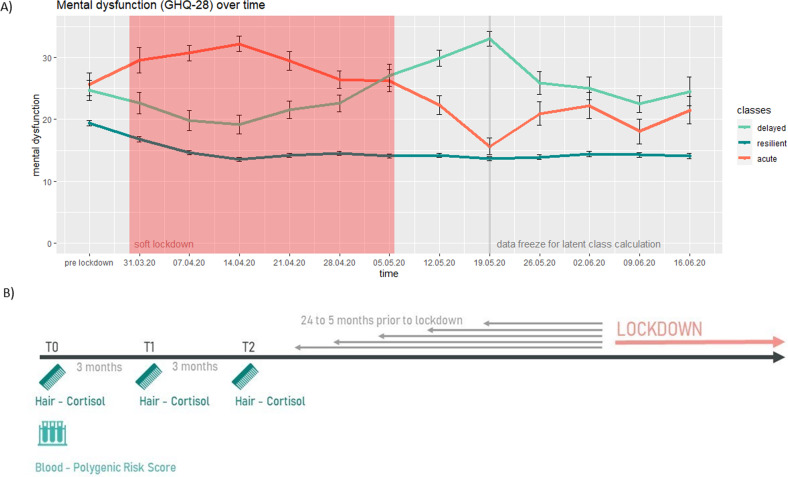
Mental health trajectories and study design. **A** Mental dysfunction of different mental health trajectories over time. Red area represents “soft” lockdown in Germany, with comparatively mild measures to restrict social contact. Calculation of latent class membership was based on their most likely latent class membership on the pre lockdown mental dysfunction value and the mental dysfunction values during the first 8 weeks of lockdown: “acute dysfunction” class = 9.0% (*n* *=* 47), “resilient” class = 82.6% (*n* = 432), “delayed dysfunction” class = 8.4% (*n* = 44) [7]. **B** Study design – bio samples collected at baseline, T1, and T1 of LORA study. Groups with different mental health trajectories were calculated based on weekly online surveys during the COVID lockdown in Germany.

With the onset of the pandemic, all participants of the original LORA study were offered the opportunity to take part in additional weekly surveys to assess their reaction to the pandemic-associated measures to restrict social contact. 523 of the 1191 LORA participants participated in the additional survey (for a detailed description of the COVID subsample, see Ahrens et al. [7]). Participants mental health was measured with the General Health Questionnaire 28 (GHQ-28), which assesses the degree of participants’ internalizing symptoms [44, 45]. Based on their mental health trajectories during the first 8 weeks of the pandemic, participants were clustered into three classes using latent growth mixture models as described in Ahrens et al. [7]. There was a larger resilient group and two smaller, vulnerable groups as described above and in the Supplemental Material. The trajectories of the classes are shown in Fig. 1A, which is adapted from Ahrens et al. [7] and extend up to 12 weeks. The classes represent typical classes found in the population after a major life event [6, 46]. The results presented here pertain only to LORA participants who opted into the additional COVID survey and additionally provided pre-pandemic hair and/or blood samples.

### Participants

Detailed inclusion criteria are described in the Supplement. Of the 523 participants who took part in the COVID surveys (for detailed descriptive analyses, see Ahrens et al. [7]), hair cortisol analyses were available from *n* = 192 participants. Genetic analyses were obtained from *n* = 364 participants, and for *n* = 142, both hair cortisol and genetic analyses were available. The descriptive demographic statistics for the three groups in the hair cortisol and the PRS datasets are shown in Tables 1–2, the descriptive demographic statistics for the complete dataset are shown in Supplementary Table 1. All participants provided written informed consent. The study meets the terms with the Code of Ethics of the World Medical Association (Declaration of Helsinki; Rickham, 2013) and was accepted by the respective Ethics Committees in Frankfurt am Main (registration number: 244/16) and Mainz (registration number: 837.105.16(10424)), Germany.

**Table 1:** Demographics of individuals with hair cortisol data *N* = 192.

Variable		Delayed Dysfunction	Resilient	Acute Dysfunction	Test statistic	*p*
		*M* ± *SD*/ frequency	*M* ± *SD*/ frequency	*M* ± *SD*/ frequency		
N		28 (14.58%)	132 (68.75%)	32 (16.67%)		
Sex	♀	25(13.02%)	104 (54.17%)	29 (15.10%)		
	♂	3 (1.56%)	28 (14.58%)	3 (1.56%)	Fisher’s exact test	0.2057
Age		32.9 (9.25)	32.6 (9.01)	28.3 (5.23)	*H*(2) = 4.18	0.1237
Marital status (baseline lockdown)	Nonmarried	9 (4.69%)	45 (23.44%)	8 (4.17%)		
	Married	5 (2.60%)	30 (15.63%)	3 (1.56%)		
	Permanent relationship	14 (7.29%)	55 (28.65%)	16 (8.33%)		
	Separated/divorced		1 (0.52%)	1 (0.52%)		
	Other		1 (0.52%)	4 (2.08%)	χ(8) = 19.91	0.0107
Number of persons living in the household		2 (0.90)	2.20 (0.93)	2.14 (0.79)	*H*(2) = 1.01	0.6022
Employment status*	Full time	12 (6.25%)	53 (27.60%)	15 (7.81%)		
	part-time	2 (1.04%)	25 (13.02%)	3 (1.56%)		
	Self-employed		3 (1.56%)			
	Parental leave		1 (0.52%)			
	Unemployed			1 (0.52%)		
	Full-time study/training	13 (6.77%)	45 (23.44%)	10 (5.21%)		
	retired					
	Other/ no answer		22 (11.46%)	3 (1.56%)	χ(12) = 13.11	0.3611
Life Events T0-T2 pre pandemic	LE	3.49 (2.49)	3.68 (1.88)	33.71 (1.66)	*H*(2) = 1.09	0.5797
GHQ last measure pre lockdown	GHQ	26 (10.30)	19.1 (8.12)	26.2 (13.50)	*F*(2) = 11.15	< 0.001

**Table 2:** Demographics of individuals with PRS data *N* = 364.

Variable		Delayed Dysfunction	Resilient	Acute Dysfunction	Test statistic	*p*
		*M* ± *SD*/ frequency	*M* ± *SD*/ frequency	*M* ± *SD*/ frequency		
N		34 (9.34%)	298 (81.87%)	32 (8.79%)		
Sex	♀	23 (6.32%)	192 (52.75%)	29 (7.97%)		
	♂	11 (3.02%)	106 (29.12%)	3 (0.82%)	Fisher’s exact test	0.0068
Age		32.8 (8.24)	32.6 (8.44)	27.4 (4.73)	*H*(2) = 11.00	0.0041
Marital status (baseline lockdown)	Nonmarried	11 (3.02%)	86 (23.63%)	8 (2.20%)		
	Married	8 (2.20%)	81 (22.25%)	2 (0.55%)		
	Permanent relationship	15 (4.12%)	117 (32.14%)	18 (4.95%)		
	Separated/divorced		3 (0.82%)	1 (0.27%)		
	Other		11 (3.02%)	3 (0.82%)	χ(8) = 12.97	0.1130
Number of persons living in the same household		1.88 (0.77)	2.21 (0.85)	2.17 (0.89)	*H*(2) = 4.52	0.1043
Employment status	Full time	16 (4.40%)	126 (34.62%)	11 (3.02%)		
	part-time	3 (0.82%)	48 (13.19%)	4 (1.10%)		
	Self-employed		10 (2.75%)			
	Parental leave		5 (1.37%)			
	Unemployed	1 (0.27%)	2 (0.55%)			
	Full-time study/ training	14 (3.85%)	85 (23.35%)	14 (3.85%)		
	retired					
	Other/ no answer		22 (6.04%)	3 (0.82%)	χ(12) = 13.55	0.3307
Life Events T0-T2 pre pandemic	LE	3.25 (2.42)	3.63 (2.00)	4.12 (1.87)	*H*(2) = 4.50	0.1054
GHQ last measure pre lockdown	GHQ	24 (11.20)	19.2 (8.81)	25.1 (11.90)	*F*(2) = 8.78	<0.001

### Polygenic risk score calculation

DNA from blood was collected from the original LORA study at baseline assessments and was used to perform Polygenic Risk Score calculations for participants from the LORA-COVID subsample. To calculate a PRS, the amount of trait-associated alleles in individuals are weighted by per-allele effect sizes derived from previously published large GWAS and normalized by a relevant population distribution [47]. Therefore, genotyping of the LORA study participants was performed in *n* = 959 participants, using an Illumina GSA-MD V 1.0 DNA Bead Array at the Broad Institute in Cambridge, Massachusetts, USA. All participants underwent quality control using PLINK v1.9 [48]. The SNPs were filtered applying the following quality control criteria: minor allele frequency ≤ 0.01, not uniquely mappable or tri-allelic variants, calling rate of ≤ 0.98 and Hardy-Weinberg-Equilibrium deviations (*p* < 1 × 10^−6^). Participants with heterozygosity rate > 0.2, missingness > 0.02, and sex mismatch were excluded from our study. Participants with a cryptic relationship with a pi-HAT > 0.2 were excluded. Filtering for population structure and criteria was performed using the following criteria: HWE *p* < 0.02, missingness = 0, MAF > 0.2, and a pruned SNP set (*r*² = 0.1, i.e. if among any pair of SNPs across the dataset these pairs surpass the linkage disequilibrium threshold, the first marker will be removed from the dataset). Using principal component analysis (PCA) we ruled out hidden population stratification using the criteria: exclusion of subjects with *SD* > 6 among the first 20 principal components (see Supplementary Fig. 1). In total, 59 participants were excluded due to the above-mentioned quality control steps from the LORA cohort (final *n* = 897). Out of 897 (after QC) participants of the LORA cohort, 364 participants who also participated in the LORA-COVID study had available genetic information for further analysis. PRS calculation was performed using the PRSice software version 2.3.1.e with default options [clump-kb 250, clump-p 1.0, clump r2 0.1, interval 5e-05, lower 5e-08, stat OR] [49]. We calculated all PRSs using the following base file data from the following GWAS and the pT thresholds with the highest variance explained, respectively: 1) ADHD, pT = 0.1; [32], 2) Bipolar Disorder, pT = 0.01; [37] 3) Alcohol Dependence, pT = 0.01; [33] 4) Anorexia nervosa, pT = 0.05; [34] 5) Anxiety disorder, pT = 0.1; [35], 6) Autism Spectrum Disorder, pT = 0.5; [36], 7) Major depression, pT = 0.3; [38], 8) Obsessive-Compulsive Disorder, pT = 0.1; [39], 9) Opioid Dependence, pT = 1.0; [40], 10) Posttraumatic Stress Disorder, pT = 0.2; [41], 11) Schizophrenia, pT = 0.05; [42], 12) Neuroticism, pT = 0.1 [43]. All base files were filtered for minor allele frequencies ≤0.01 and INFO score filtering (INFO > 0.8). There was no participant overlap between the present study sample and the used base files from the different studies. PRS calculation was performed using age, sex, and the first five principal components for population stratification as covariates. All PRS were z-transformed prior to further processing.

After PRSs calculation, an exploratory bifactor analysis with Schmid-Leiman transformation was performed for dimensionality reduction [50, 51]. For a detailed description of the assumptions of a bifactor model see Supplemental Material.

### Hair cortisol concentration calculation

Hair samples were collected quarterly in the LORA sample to determine glucocorticoid hair cortisol concentration and capture the individual’s potential environmental risk as well as physiological differences. As illustrated in Fig. 1B, the first 3 HCC values from the original LORA study were obtained for each participant. Because participants were continuously enrolled in the original LORA study from the beginning of 2017 to the end of 2019, the last hair sample for individual participants ranged from 5 to 27 months prior to the pandemic outbreak in each case. Since it has also been demonstrated that HCC shows a strong trait component and state influences are lower [9], we averaged the cortisol levels over the first three measurements of the original LORA study, spanning a time of nine months and interpreted these as trait-like baseline levels.

For HCC calculation two hair strands of at least 3 cm length, corresponding to 10 mg of hair, were cut as close to the scalp as possible from a posterior vertex position. Hair cortisol analyses were carried out by the Laboratory from Prof. Clemens Kirschbaum (DRESDEN LAB SERVICE GMBH). Here, strands were cut into 3 cm segments. In line with the protocol of Davenport et al. [52], hair was washed and steroids extracted. Therefore, each hair segment was put into a 10 ml glass container, then 2.5 ml isopropanol was added, and the tube gently mixed on an overhead rotator for three minutes. After decanting, the wash cycle was repeated two times. Then the hair samples were allowed to dry for at least 12 hours. Next, the hair segments were weighed out and 7.5 mg were transferred into a 2 ml cryo vial. 1.5 ml of pure methanol was added and the steroid extraction was performed for 18 hours. Samples were then spun in a microcentrifuge at 10.000 rpm for 2 min, and 1 ml of the clear supernatant was transferred into a new 2 ml glass vial. The alcohol was evaporated at 50 degrees Celsius under a constant stream of nitrogen until the samples were completely dried. Finally, 0.4 ml of water was added and the tube vortexed for 15 sec. Fifty microliters were removed from the vial and used for cortisol determination with a commercially available immunoassay with chemiluminescence detection (CLIA, IBL-Hamburg, Germany). The intraassay and interassay coefficient of variance of this assay is below 8%. HCC were checked for extreme outliers (3rd quartile + 3*interquartile range; 1st quartile – 3*interquartile range) and log- and z-transformed prior to further processing. The intraclass correlation coefficient of the three measurements at an interval of 3 months based on mean-rating (k = 3), absolute agreement, 2-way mixed-effects model showed moderate to good reliabilities [53] (ICC = 0.76, *CI* = [0.69; 0.82], *F*(147,291) = 4.22, *p* < 0.001). These results are consistent with the high test-retest reliabilities of 0.68 to 0.79 reported by Stalder and Kirschbaum [9].

### Statistical Analysis

For a detailed description of the statistical analyses, included covariates and power analyses see Supplemental Material.

## Results

Means, standard deviations, and correlations with confidence intervals of the PRS are shown in Supplementary Table 2.

### Bifactor model of genetic risk factors

Kaiser-Meyer-Olkrin measure, Bartlett’s test of sphericity and Eigenvalue calculation verified the sampling adequacy for factor analysis (see Supplemental Material for results). An explorative bifactor analysis with Schmid-Leiman transformation was calculated. The model fit the data well with χ²(24) = 20.36, *p* < 0.68, BIC = −121.17, RMSR = 0.02.RMSEA < 0.01, CI10%[0;0.04], with a reliability of the general factor and the sub-factors of Omega total = 0.61; Alpha = 0.51.

**Fig. 2:**
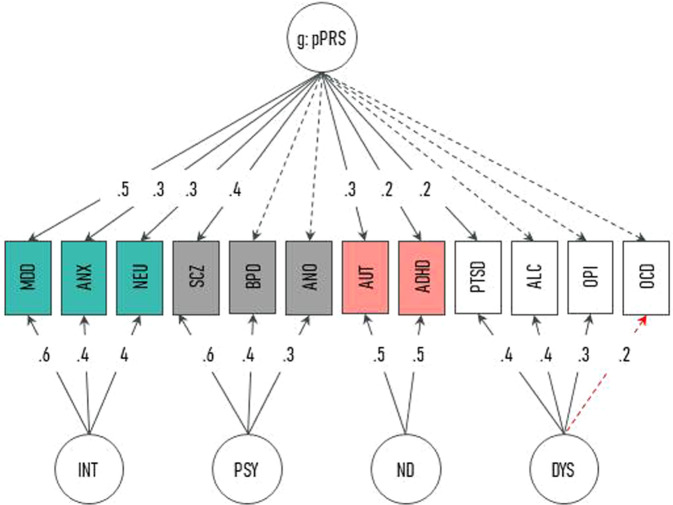
Bifactor model omega with Schmid-Leiman transformation of the 12 genetic risk factors. Factor 1: “INT” = internalizing disorders; factor 2: “PSY” = psychotic disorders; factor 3: “ND” = neurodevelopmental disorders; factor 4: “DYS” = dysfunctional coping disorders; “g”: general pleiotropic pPRS factor. Dotted arrows = loadings < 0.20; black arrows = positive loadings; red arrow = negative loading.

The bifactor model with 4 factors based on our 12 PRSs and a general factor g (general pleiotropic pPRS factor), with Schmid-Leiman factor loadings greater than 0.2, is displayed in Fig. 2. Factor 1 called “INT” consisted of internalizing disorders and an associated personality trait (MDD, ANX, NEU). Factor 2 named “PSY” was characterized by disorders with mainly psychotic features and Anorexia nervosa (SCZ, BPD, ANO). Factor 3 titled “ND” was expressed by childhood-onset neurodevelopmental disorders (ADHD, AUT). Factor 4 named “DYS” was designated to dysfunctional coping disorders and the opposite of controlled behavior (PTSD, ALC, OPI, negatively OCD). The general pleiotropic pPRS factor loaded on all PRSs (with loadings over 0.2 on MDD, ANX, NEU, SCZ, AUT, ADHD, PTSD).

### Main effects of genetic risk and long-term cortisol secretion

Results showed a significant main effect of long-term HPA axis activation for the association with the acute dysfunction class compared to the resilient class. Specifically, elevated HCC during a time period of 9 months significantly predicted whether a participant belonged to the acute or the resilient class, *b* = 0.45, *p* = 0.045; odds = 1.56, Nagelkerke *R*^2^ = 0.25. The effects remained stable even when controlling for the usage of hormonal contraceptives, hair treatment, or time interval between hair sample collection and lockdown, and mental health at the last measurement point before lockdown (see Supplementary Table 3 for sensitivity analyses). There was no significant association of HCC with the delayed dysfunction class compared to the resilient class.

**Fig. 3:**
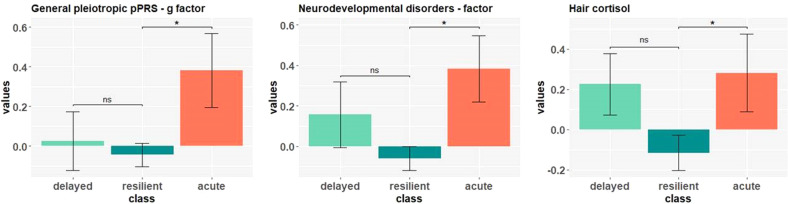
Class comparisons regarding significant environmental and genetic factors. Significance based on bivariate logistic regressions (reference class = “resilient”). Data is z-transformed. Error bars = +/− 1 standard error (SE). “Delayed” = “delayed dysfunction” class; “acute” = “acute dysfunction” class.

At genetic level, there was a significant association of the acute class with the general pleiotropic pPRS factor, where a higher genetic load predicted the class membership, *b* = 0.44, *p* = 0.025, odds ratio = 1.55, Nagelkerke *R*^2^ = 0.21. The delayed dysfunction class showed no significant association of the general pPRS factor when compared with the resilient class. Similarly, as exploratorily tested, the combined genetic risk factor for ADHD and ASD was associated with the acute class compared with the resilient class, with a higher genetic risk of individuals in the acute class, *b* = 0.43, *p* = 0.031, odds ratio = 1.53, Nagelkerke *R*^2^ = 0.21. This association again was not significant for the delayed dysfunction class compared to the resilient class. The main effects of genetic risk and long-term cortisol secretion as potential environmental risk factor are displayed in Table 3. The observed effects remained significant even when controlling for age, gender, and mental health at the last measurement point before lockdown (see Fig. 3 for significant class comparisons). In the explorative analyses none of the risk groups were associated with the PRS-factors “internalizing disorders”, “psychotic disorders”, and “dysfunctional coping disorders”.

**Table 3:** Multiple bivariate logistic regressions: Main effects of genetic risk and long-term cortisol secretion.

Variable	Delayed Dysfunction vs. Resilient					Acute Dysfunction vs. Resilient				
	Estimate	*CI*	*p*	odds	*R*²	Estimate	*CI*	*p*	odds	*R*²
*N* = *192*										
HCC	0.34	−0.13;0.84	0.161	1.41	0.16	**0.45**	**0.02;0.91**	0.045*	**1.56**	**0.25**
*N* = *364*										
g-factor	0.11	−0.26;0.49	0.549	1.12	0.05	**0.44**	**0.06;0.82**	0.025*	**1.55**	**0.21**
INT-factor	−0.16	−0.53;0.20	0.383	0.85	0.05	0.18	−0.21;0.57	0.361	1.19	0.18
PSY-factor	0.16	−0.20;0.52	0.394	1.17	0.05	0.00	−0.38;0,38	0.994	1.00	0.18
ND-factor	0.25	−0.12;0.61	0.185	1.28	0.06	**0.43**	**0.04;0.82**	0.031*	**1.53**	**0.21**
DYS-factor	0.10	−0.26;0.46	0.574	1.11	0.05	0.30	−0.06;0.66	0.099	1.35	0.19

The main effects of bivariate logistic regressions are shown. Bivariate logistic regressions regarding the main effect of hair cortisol were calculated in a sample of *n* = 192 participants. Bivariate logistic regressions regarding the main effects of genetic risk factors were calculated in a sample of *n* = 364 participants. All bivariate logistic regressions were controlled for age, sex, and pre-lockdown mental health status. R² = Nagelkerke pseudo R². The hair cortisol analysis also took into account the time interval between the hair sample collection and the pandemic. *HCC* hair cortisol concentration, *INT* internalizing disorders, *PSY* psychotic disorders, *ND* neurodevelopmental disorders, *DYS* dysfunctional coping disorders; *g* general pleiotropic pPRS factor. Bold indicates bivariate logistic regression is significant.

### Interaction effects of genetic risk and long-term cortisol secretion

When integrating both genetic risk factors and HCC in one analysis to predict the membership to the acute dysfunction class, compared to the resilient class, we found neither a significant interaction effect of HCC with the general pleiotropic pPRS factor (*b* = −0.17, *CI* = [−0.84;0.44], *p* = 0.593) on class nor a significant interaction of HCC with the factor neurodevelopmental disorders (*b* = 0.05, *CI* = [−0.48;0.66], *p* = 0.846). Nonetheless, the main effect of the potentially environmental and physiological factor HCC remained significant and stable, also when controlling for the covariates age, sex, mental health at the last measurement time point before the pandemic, hormonal contraception, hair treatment, and time interval of hair sampling to the pandemic. Means, standard deviations, and correlations with confidence intervals of the genetic risk factors and HCC are shown in Supplementary Table 4. See Supplementary Tables 5-6 for the results on the interaction analyses.

## Discussion

The present study investigated the utility of prospectively sampled biological parameters to identify individuals belonging to a group of participants with deteriorated mental health after the outbreak of the COVID-19 pandemic in Germany. The largest group of participants showed a stable mental health trajectory, as shown in previous work [7], and served as a reference class herein. The likelihood of belonging to one of the two smaller groups, which showed either an acute dysfunction or a delayed dysfunction, was estimated. For risk prediction, one overall genetic risk factor and four sub-factors extracted from a bifactor model of the genetic risk for 12 different psychiatric disorders in combination with HCC, a highly heritable indicator of long-term cortisol secretion and a potential indicator of environmental stress and physiological risk, were used. Overall, the results suggest an increased likelihood for individuals with higher values on the general pleiotropic pPRS to be assigned to the acute dysfunction class rather than to the resilient class. The same holds true for the association with HCC and for the exploratorily tested genetic risk factor for childhood-onset neurodevelopmental disorders (ND). Participants with higher HCC, or with higher values on the “ND” factor, were more likely to belong to the acute dysfunction class compared to the resilient class. Neither factor had an effect on the risk for belonging to the delayed dysfunction class compared to the resilient class.

### Genetic risk factors

Regarding the genetic risk for a comprehensive set of the most common psychiatric disorders and a related personality trait, a bifactor model was calculated that described the data well, as indicated by the fit indices. The resulting five factors entailed four sub-factors and one general factor. The composition of the extracted factors is not a main hypothesis of this study, therefore a detailed description of the factors and contextualization in the literature can be found in the Supplemental Material. The found factors in the current study are in line with previous studies. It has already been shown that mental disorders differentially share variance with one common genetic factor beyond the influence of disease-specific factors [27, 28, 54]. E.g., Grotzinger et al. showed that 11 major mental disorders share variance with four factors and can be calculated in addition to a general factor [29]. The four broad factors (Neurodevelopmental, Compulsive, Psychotic, and Internalizing) postulated by Grotzinger et al. [29] are of similar content and support the factor structure found in the current study.

Out of the five factors identified in this study, the general pleiotropic pPRS factor and the exploratorily tested neurodevelopmental disorders factor distinguished between the acute dysfunction and resilient class membership, but not between the delayed dysfunction and resilient class membership. Accordingly, the hypothesis that the vulnerable subjects of the risk groups “acute dysfunction” and “delayed dysfunction” both have a generally increased genetic risk for developing a psychiatric disorder could only partially be confirmed. It can be hypothesized why the general factor of genetic risk, as well as the “ND” factor, are suitable for the distinction between the groups, while the other risk factors do not provide any explanatory meaning. Hypothetically speaking, the effect may be explained by impaired emotion regulation and irritability. A detailed discussion of the exploratorily discovered “ND“ factor for distinguishing the acute dysfunction and resilient group can be found in the Supplemental Material under the section “Discussion of extracted factors”

Research underlines the fact that PRSs currently have no clinical utility because effects are too small and PRS do not serve well for risk prediction [55]. Here, the factors of our bifactor model suggest that using compound PRSs might be a promising approach, however, the predictive power does not seem to improve beyond single PRS. Nevertheless, this approach fits the dimensional concept of psychiatric disorders, which understands them on a continuum [56]. Thus, our approach is in line with the work by Caspi, who postulated that biomarkers related to the general risk for a psychiatric disorder should be identified first [28]. In a second step, the identification of general risk factors should be followed by an examination of specific genetic risk factors for particular disorders [28]. This approach takes into account the pleiotropic mechanisms between different mental disorders. Hence, associations with a general pleiotropic factor may be found that cannot be identified at the individual level of diagnosis-specific PRS.

### Chronic HPA axis activation as a potential risk factor

Considering the HCC effect, we found similar results as for genetic risk factors. Again, participants with an acute deterioration in mental health in reaction to the pandemic showed significantly higher HCC prior to the pandemic compared to resilient participants. The results indicate that individuals who tend to react directly within one month to an external stressor generally show an increased long-term HPA axis activity. One explanation could be that participants of the acute dysfunction class may have experienced prolonged environmental stress (e.g., quarrels in the family or at work). This chronic stress may have led to long-term overactivity of the HPA axis and may have resulted in relatively increased exposure to cortisol, negatively impacting mental health. However, all of our cortisol levels were within the range reported by other studies with healthy participants [57]. Another explanation would be physiological differences that result in an increased endogenous cortisol release rate, even though they had the same amount of stress exposure as the participants belonging to the resilient class. Because of inherited increased HPA activation they might have had a relatively increased secretion of cortisol. HCC is proven to have a more substantial trait than a state component [9], which can be interpreted as a physiological predisposition to cortisol secretion. It has already been shown that HPA regulation is heritable to a non-negligible extent. Twin studies suggest that cortisol secretion is up to 62% heritable [58]. In vervet monkeys, the genetic contribution to individual differences in HCC was shown [59]. In addition, an initial study in humans showed that the heritability of HCC is reported to be as high as 72% [4]. In combination with earlier evidence on the association of HCC with diurnal cortisol secretion [16, 60], it is possible that participants with higher HCC, due to genetic predispositions or experienced prolonged environmental stress, are stressed more easily or react more physiologically. Accordingly, this would also explain why they responded stronger and faster to the pandemic and associated socio-political restrictions than participants with less HPA axis activation. Moreover, we expected similar results for the comparison between the delayed dysfunction and the resilient group, but this hypothesis was not confirmed. A replication of the current study with larger risk groups and therefore improved power could provide further insights. Notably, the lack of a significant difference in HCC between the delayed dysfunction and the resilient group is not equal to an actual absence of a group difference in the trait release of endogenous cortisol. Regarding the previously calculated minimum effect size required for detecting a significant difference in our sample with a power of 0.80, it can be concluded, that the actual effect is probably smaller than OR = 1.68.

These results are in line with other studies that reported increased HCC in individuals with objectively stressful living conditions, such as shift workers, pain patients, hospitalized infants, and students who experienced major live events [10–14]. However, further research needs to be pursued to target the utility of HCC as a stress marker.

### Interaction of HCC and PRSs

We did not detect a significant interaction between HCC and genetic risk. We had hypothesized to see an interaction based on the trend found by Bolhois et al., who studied the interaction of HCC and the schizophrenia PRS on brain structure [3]. In contrast, the results obtained in the current study fit with analyses by Rietschel et al., who have shown no association between the genetic risk for MDD and neuroticism with HCC [4]. This may suggests that PRSs and HCC have additive value to identify vulnerable individuals, but not represent an exponential increase in risk. At the same time, our analyses may also have been underpowered to detect a significant interaction effect. To detect a main effect with the given sample size of the complete data, a main effect of OR = 1.71 would be detectable with a power of 0.80. As pointed out by Luan et al. [61] and Musliner et al. [62], to detect the main effect of environmental factors, smaller sample sizes are required than when investigating gene x environment interactions. Accordingly, the interaction effect would have to be significantly above the calculated size to be detectable.

### Relevance

The study combined several biological and potential environmental factors in the prospective identification of individuals at increased risk for developing mental disorders. To determine an individual’s baseline level of HCC, hair strains were sampled not only prospectively but also over a considerable period of time (9 months). While other studies on prospective measures did not collect hair until after the occurrence of a major stressor, we obtained truly prospective values in the 27 to 5 months period prior to the pandemic. The study design also ensured that no psychiatric diagnosis was present at the time the first hair sample was collected. Moreover, we provided evidence of a predictive character of PRSs as summarized in a bifactor model, to classify participants with a not clinically defined trait. This approach merits further investigation. The way it condenses genetic information may be used to identify individuals’ risk for psychiatric disorders at a very early stage. Implementing preventive measures and interventions to buffer the acute stress response to environmental stressors might prove beneficial for these individuals at risk. Therefore, the found factor structure warrants replication in other independent samples.

### Limitations

The findings of this study should be interpreted in light of several limitations. Two apparent limitations are the experimental nature of the analyses and the limited sample sizes/power. In particular, participants are notably underrepresented in the at-risk groups, especially in the HCC dataset (delayed dysfunction *n* = 28, acute dysfunction *n* = 32) and complete dataset (delayed dysfunction *n* = 20, acute dysfunction *n* = 22).

Moreover, the exploratory bifactor model showed two anomalies, which require further investigation before generalizing the result. The first anomaly concerned the correlation of the g-factor with the other four sub-factors (as shown in Supplementary Table 4). The second anomaly was the phenomenon that not all PRS had loadings above 0.2 on the pleiotropic p-factor. Conceptually a bifactor model assumes the existence of a general factor on which all items (in our case PRSs) load and sub-factors that are orthogonal to the general factor and to each other. As pointed out by Eid et al. [92], those anomalies have to be expected when bifactor models are applied and are common phenomena. A structured review on studies that have applied bifactor models revealed that anomalies occurred in 61% of the studies included in the review [92]. Since it is a known problem and the model fit was good, we have not rejected the model [92]. However, the appropriateness of the bifactor model is questionable and requires further investigation. Therefore, future research should critically examine whether there is indeed an orthogonal general pleiotropic pPRS factor. Furthermore, as with many studies investigating HCC, women were overrepresented in the current study, especially in the comparatively small risk groups. This is most likely because female sex represents a risk factor for mental deterioration during the pandemic [63], and women are therefore more likely to fall into the risk trajectories. Another possible explanation for the overrepresentation of women in the current study sample is the required hair sample length of 3 cm for HCC analyses. Notably, the overall HCC effect remained significant when corrected for sex. Thus, the overrepresentation of women in the study sample does not hamper the generalizability of the findings. Also, we used GWAS, which predominantly contain individuals of European ancestry. Thus, it is possible that the bifactor model cannot be replicated in other ethnicities. Nevertheless, the class allocation of the participants was not driven by genetic heterogeneity.

In addition, the main effects were just significant, may not be very stable, and should therefore be interpreted with caution. The main effect of the ND factor discovered in the exploratory analyses did not survive correction for multiple testing. Accordingly, the results need to be replicated in larger samples. The results described in this study do not yet allow us to infer the mechanisms underlying the differences in the groups. The mechanistic underpinnings of the observed effects should be analyzed in further studies, as such explanations are beyond the scope of the present work. Since there is evidence for the trait character of the heritable marker HCC [9], it might be interesting to look at the genetic risk of elevated HCC levels. A first approach has already been made by Rietschel [4], who investigated a PRS for plasma cortisol, but to our knowledge, there is currently no suitable PRS for long-term cortisol levels. Future studies should focus on this aspect.

### Implications and conclusion

The present study provides first evidence that long-term elevated basal cortisol secretion is associated with a direct deterioration of mental health in the form of an extreme stress response during the COVID lockdown in Germany as a defined psychosocial stressor. A higher basal hair cortisol level could be indicative of a lowered threshold for a (negative) reaction towards environmental stressors and thus may represent a vulnerability factor that may also be genetic. Therefore, HCC, a general pleiotropic p factor for genetic risk, and a factor containing childhood-onset neurodevelopmental disorders may be helpful to timely identify vulnerable individuals with strong stress overreactivity for future potentially traumatic events. These individuals could be addressed by services to buffer the first extreme stress reaction and the resulting individual risk. At the same time, the determined biological factors are currently limited as general predictors, because they failed to identify another vulnerable group with a delayed reaction to the lockdown. Further research with larger sample sizes is necessary to assert whether this specificity in prediction is biologically grounded, to replicate these first indications, and to increase the validity of HCC and PRSs as biomarkers. In addition, it is also essential to examine how the two risk groups (acute dysfunction, delayed dysfunction) evolve as the pandemic progresses to draw conclusions about the long-term mental health outcomes and assess the importance of biological predictors for these groups.

In conclusion, this study provides empirical evidence that integrating biological risk factors into the diagnostic routine could support an early identification of individuals with an acute overcompensation to stressors.

## Supplementary information

Supplemental Material

## Data Availability

The datasets presented in this article are not readily available because participants of the study did not give permission to publish their genome-wide data, based on obvious conflict with General Data Protection Regulation (OJ L 119, 04.05.2016;cor. OJ L 127, 23.5.2018.; https://gdpr-info.eu/). Reasonable requests to access the data should be directed to Andreas Reif, andreas.reif@kgu.de.
