# Supplementary material for: Association of polygenic risk scores and hair cortisol with mental health trajectories during COVID lockdown
Source: Transl Psychiatry. 2022 Sep 21;12:396. doi: 10.1038/s41398-022-02165-9 (PMC9490720; doi:10.1038/s41398-022-02165-9)
Supplement: Supplementary file 1 — Supplemental Material [file 41398_2022_2165_MOESM1_ESM.docx]

**Supplemental Material**

*Study design*

The LORA study is being conducted in Frankfurt and Mainz in the Rhine-Main area of Germany. The study examines initially healthy participants after in-depth genotyping and phenotyping, which includes quarterly examinations concerning their mental health and the exposed stressors and life events via the online data collection platform SecuTrial ©.

*Further description of mental health trajectories from Ahrens et al.* ^7^

The two smaller groups showed either immediate ("acute dysfunction" class) or somewhat delayed ("delayed dysfunction" class) deterioration in mental health. Originally, after consideration of the first 8 weeks of the lockdown the in this study called “acute dysfunction” class was named "recovered", as the mental dysfunction scores improved towards the end of the observation period ^7^. Considerations of a more extended period showed that the initial adaptation and improvement was only of short duration, so the authors decided to avoid the original name for this and future publications.

*Participants*

Participants between 18 and 50 years of age were included in the LORA study. Further requirements were sufficient knowledge of German and normal or corrected vision. Only participants without any lifetime diagnoses of organic mental disorders, or schizophrenia, or bipolar disorder, or substance dependence syndromes, as well as any further existing severe axis-I disorder or medical conditions, could be included ^31,64^.

*Bifactor Model*

The bifactor model consists of a general factor that loads on all PRSs directly and four grouping factors that load on sub-groups of the PRSs ^50^. Thereby each PRS can load on the grouping factors as well as on the general factor. The grouping factors are orthogonal, i.e. uncorrelated with the general factor^50^. Bifactor models are used when research pays as much attention to the subgroup domains as to the general factor ^51^. Therefore the subdomains can be used independently from the general factor to predict external variables ^51^, as our class membership.

*Covariates*

In all analyses, we controlled for age and sex, as well as mental health measured at the last assessment time point prior to the soft lockdown in Germany (between 4 and 1 month(s) prior to the start of the restrictions, depending on the individual assessment schedule) using the general health questionnaire assessed with 28 items (GHQ-28) ^44,45^.

In addition, all analyses that included HCC were controlled for the time interval between last hair sampling and the start of the lockdown. Moreover, they were controlled for hormonal contraception (pill, contraceptive ring, IUD) and for hair treatment (e.g. hair dyeing or perming) at the time of hair sample collection.

*Statistical Analysis*

Data analysis was performed using R (Version 4.0.5, 2021-03-31) and for power analyses G*Power 3.1.9.4 was used. Significance was tested at a 5% level. A bifactor model was calculated using the *omega()* function of the *psych* package^65^. This function estimates an exploratory factor analysis, does principal axes factoring, following a Schmid-Leiman transformation, finds the general factor loadings, and finally estimates ω ^66,67^. The Schmid-Leiman transformation provides the factor scores for the general factor and the subgroup factors. To check how well the model represents the data, the omega function offers different fit indices: The Bayesian information criteria (BIC), Root Mean Square Error of Approximation (RMSEA), the Standardized Root Mean Square Residual (SRMR), as well as the goodness of fit statistic (χ²). As described by Hu and Bentler, a good fit is indicated by RMSEA < .06 and SRMR < .08 ^68^. The BIC smaller values indicate better model fit ^69^, and the χ²-test should be insignificant to accept the null hypothesis that the model describes the data well.

Bivariate logistic regressions were calculated to determine whether higher genetic risk factors were associated with an increased likelihood of being a member of a vulnerable class (acute and delayed dysfunction), controlling for age, sex, and mental health status prior to COVID-lockdown (assessed on the GHQ-28). The "resilient" class served as a reference class.

Accordingly, the analyses were repeated twice for each latent factor: (1.) comparison of the "acute dysfunction" class with the "resilient" class and (2.) comparison of the "delayed dysfunction" class with the "resilient" class. The same procedure was used to analyze the hair cortisol effects, also controlling for sample collection time, and for the interaction effects of HCC and genetic risk factors. To this end, a total of 12 bivariate logistic regressions was conducted to explore the question whether HCC or each of the (5) latent PRS-factors significantly predicted membership to the acute dysfunction vs resilient, or delayed dysfunction vs resilient class. Regarding the given sample sizes, a logistic regression with 364 participants in the PRS dataset would be sensitive to effects of odds ratio = 1.45 with 80% power (alpha = .05, two-tailed). With identical assumptions and a given sample size of 192 participants in the hair cortisol dataset the analyses would be sensitive to effects of odds ratio = 1.68.

*Assumptions factor analysis*

Kaiser-Meyer-Olkrin measure verified the sampling adequacy for factor analysis KMO = .63. Except for the KMO value of the PRS for alcohol dependence (KMO = .47), all other KMO values for the PRS were > .53, therefore above the acceptable cutoff of .5 ^70^. The correlations between the PRSs were sufficiently large for the analysis, indicated by Bartlett's test of sphericity χ²(66) = 313.72, *p* < .001. Prior to calculating a bifactor model, we ran an initial analysis to obtain Eigenvalues. Four components had Eigenvalues over Kaiser's criterion of 1. To account for the bifactorial structure of mental dysfunctions ^28,29^.

*Discussion of extracted factors*

The first factor “INT” was characterized by internalizing disorders and an associated personality trait. This outcome is in line with previous studies using a bifactorial model, showing that MDD and generalized anxiety disorder underlie a common general factor ^71^. Also, the relationship between internalizing disorders and neuroticism in this common factor is plausible. Neuroticism has previously been associated with anxiety in MDD ^72^ and proven to be predictive for the onset of unipolar affective disorders and anxiety disorder as well as for the comorbid occurrence of both disorders ^73^. The second factor “PSY” combines disorders with psychotic symptoms. Both, schizophrenia and bipolar disorders are usually accompanied by psychotic symptoms ^56^. Further, psychotic symptoms can appear in anorexic patients as well ^74^, and genetic factors overlap to a certain extent in schizophrenia and anorexia nervosa ^75^. The third factor “ND” consists of childhood-onset neurodevelopmental disorders. This compound factor can be expected because the two disorders often co-occur and share common genetic factors ^76^. Moreover, not only the time of disorder onset is similar, but also the symptoms of ADHD and ASD often overlap ^77^. The “ND” factor, may be suitable for the distinction between the groups, while the other risk factors do not provide any explanatory meaning, as one of the core symptoms of ADHD is the difficulty of regulating emotions triggered by external stimuli ^78^. As such, the pandemic can be interpreted as an external stimulus that could certainly trigger an immediate deterioration of mental health. Hypothetically speaking, people who are more irritable, such as patients diagnosed with ASD ^79^, react faster to an external stressor and a change in their daily routine. Moreover, ADHD patients often show executive dysfunctions ^80^, such as time structuring difficulties, as well as patients diagnosed with ASD ^81^. External structuring decreased to some extent during the pandemic, which may have intensified the difficulties and strain for genetically vulnerable subjects. This may explain why this factor, in particular, is suitable for the distinction between the classes. In addition, it provides a possible explanation why the group comparison between the acute dysfunction class vs. the resilient class became significant, while the comparison between the delayed dysfunction class and the resilient class did not: Those subjects who may be genetically prone to a very rapid response to an external stressor were also most likely to show an acute, but not a delayed response during the pandemic. The fourth factor “DYS” encompasses dysfunctional coping disorders and the inverse of controlled behavior. Prior studies support this finding of a shared molecular genetic basis for PTSD and ALC ^82^. Furthermore, ALC, OPI, and OCD share the common feature of exhibiting involuntary repetitive behaviors, which can negatively affect personal and work life for those affected. In addition, loss of control appears to play a critical role in this factor. ALC and OPI incorporate loss of control over the corresponding drug ^83,84^ assumed to occur along with maladaptive emotion regulation. The finding that OCD enters inversely into the factor may also confirm that loss of control plays a crucial role. Speculatively, PTSD patients also lack control over their trauma (i.e., intrusions). In addition, the so-called general pleiotropic pPRS factor was identified with loadings on all PRSs. This aligns with the dimensional concept of psychiatric disorders ^53^.

**Supplemental Figures**


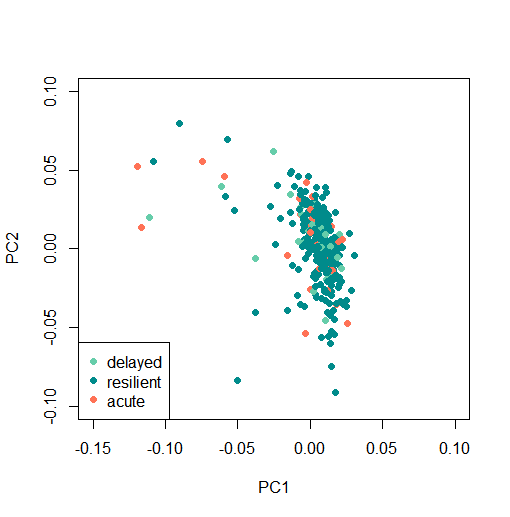


*Supplementary Fig. 1*. Genetic PCA plot of the PRS data sample (n =364) illustrates genetic homogeneity of the sample across the frist two components which is independent of the respectively allocated class of the participants.

**Supplemental Tables**

Supplementary Table 1. *Demographics of complete data N = 142.*

|  |  | Delayed Dysfunction | Resilient | Acute Dysfunction |  |  |
| --- | --- | --- | --- | --- | --- | --- |
| Variable |  | *M* ± *SD* / frequency | *M* ± *SD* / frequency | *M* ± *SD* / frequency | Test statistic | p |
|  |  | 20 (14.08%) | 100 (70.42%) | 22 (15.49%) |  |  |
| Sex | ♀ | 19 (13.38%) | 79 (55.63%) | 20 (14.08%) |  |  |
|  | ♂ | 1 (0.70%) | 21 (14.79%) | 2 (1.41%) | Fisher's exact test | .1528 |
| Age |  | 32.15 (8.59) | 32.74 (8.76) | 28.59 (5.04) | H(2) = 2.72 | .257 |
| Marital status  (baseline lockdown) | Non-married | 7 (4.93%) | 36 (25.35%) | 5 (3.52%) |  |  |
|  | Married | 3 (2.11%) | 22 (15.49%) | 2 (1.41%) |  |  |
|  | Permanent relationship | 7 (4.93%) | 36(25.35%) | 5 (3.52%) |  |  |
|  | Separated/divorced |  |  | 1 (0.70%) |  |  |
|  | Other |  | 1 (0.70%) | 3 (2.11%) | χ(8) = 19.61 | .01192 |
| Number of persons living in the same household |  | 1.9 (0.79) | 2.15 (0.91) | 2.05 (0.78) | H(2) = 1.14 | .5648 |
| Employment status* | Full time | 8 (5.63%) | 41 (28.87%) | 10 (7.04%) |  |  |
|  | part time | 2 (1.41%) | 19 (13.38%) | 2 (1.41%) |  |  |
|  | Self-employed |  | 3 (2.11%) |  |  |  |
|  | Parental leave | 1 (0.70%) |  |  |  |  |
|  | Unemployed |  |  |  |  |  |
|  | Full-time study/training | 10 (7.04%) | 32 (22.54%) | 7 (4.93%) |  |  |
|  | retired |  |  |  |  |  |
|  | Other/ no answer |  | 5 (3.52%) | 3 (2.11%) | χ(8) = 8.32 | .40257 |
| Life Events baseline T0-T2 pre pandemic | LE | 3.59 (2.69) | 3.56 (1.82) | 3.77 (1.66) | H (2) = 0.61 | .7378 |
| GHQ last measure pre lockdown | GHQ | 26.0 (12.1) | 18.6 (7.36) | 26.5 (13.1) | F(2) = 10.2 | < .001 |

Supplementary Table 2. *Means, standard deviations, and correlations with confidence intervals of the genetic risk and HCC.*

| Variable | ADHD PRS | ALC PRS | ANO PRS | ANX PRS | AUT PRS | BPD PRS | MDD PRS | NEU PRS | OCD PRS | OPI PRS | PTSD PRS | SCZ PRS |
| --- | --- | --- | --- | --- | --- | --- | --- | --- | --- | --- | --- | --- |
| ADHD PRS |  |  |  |  |  |  |  |  |  |  |  |  |
|  |  |  |  |  |  |  |  |  |  |  |  |  |
| ALC PRS | .02 |  |  |  |  |  |  |  |  |  |  |  |
|  | [-.08, .12] |  |  |  |  |  |  |  |  |  |  |  |
|  |  |  |  |  |  |  |  |  |  |  |  |  |
| ANO PRS | .01 | -.06 |  |  |  |  |  |  |  |  |  |  |
|  | [-.09, .12] | [-.16, .04] |  |  |  |  |  |  |  |  |  |  |
|  |  |  |  |  |  |  |  |  |  |  |  |  |
| ANX PRS | .03 | .05 | .06 |  |  |  |  |  |  |  |  |  |
|  | [-.07, .14] | [-.05, .15] | [-.04, .16] |  |  |  |  |  |  |  |  |  |
|  |  |  |  |  |  |  |  |  |  |  |  |  |
| AUT PRS | .32** | .04 | .10 | .09 |  |  |  |  |  |  |  |  |
|  | [.22, .41] | [-.06, .14] | [-.00, .20] | [-.01, .19] |  |  |  |  |  |  |  |  |
|  |  |  |  |  |  |  |  |  |  |  |  |  |
| BPD PRS | -.07 | -.00 | .10 | .04 | .04 |  |  |  |  |  |  |  |
|  | [-.17, .03] | [-.10, .10] | [-.00, .20] | [-.06, .14] | [-.06, .14] |  |  |  |  |  |  |  |
|  |  |  |  |  |  |  |  |  |  |  |  |  |
| MDD PRS | .15** | -.03 | .06 | .35** | .16** | .03 |  |  |  |  |  |  |
|  | [.05, .25] | [-.13, .07] | [-.05, .16] | [.26, .44] | [.06, .26] | [-.07, .14] |  |  |  |  |  |  |
|  |  |  |  |  |  |  |  |  |  |  |  |  |
| NEU PRS | .13* | -.02 | .05 | .22** | .01 | .05 | .34** |  |  |  |  |  |
|  | [.03, .23] | [-.12, .08] | [-.05, .15] | [.12, .31] | [-.09, .11] | [-.06, .15] | [.24, .43] |  |  |  |  |  |
|  |  |  |  |  |  |  |  |  |  |  |  |  |
| OCD PRS | .06 | -.12* | .01 | -.01 | .09 | .02 | -.03 | .01 |  |  |  |  |
|  | [-.04, .16] | [-.22, -.02] | [-.09, .12] | [-.11, .09] | [-.01, .19] | [-.09, .12] | [-.13, .08] | [-.09, .12] |  |  |  |  |
|  |  |  |  |  |  |  |  |  |  |  |  |  |
| OPI PRS | .01 | .09 | .08 | .05 | .03 | .08 | .08 | .08 | -.04 |  |  |  |
|  | [-.09, .11] | [-.01, .19] | [-.02, .18] | [-.05, .15] | [-.07, .13] | [-.02, .18] | [-.02, .18] | [-.03, .18] | [-.14, .06] |  |  |  |
|  |  |  |  |  |  |  |  |  |  |  |  |  |
| PTSD PRS | .09 | .15** | .11* | .03 | .08 | .16** | .13* | .03 | -.03 | .18** |  |  |
|  | [-.01, .19] | [.05, .25] | [.01, .21] | [-.07, .13] | [-.02, .18] | [.06, .26] | [.03, .23] | [-.07, .14] | [-.13, .07] | [.08, .28] |  |  |
|  |  |  |  |  |  |  |  |  |  |  |  |  |
| SCZ PRS | .05 | -.01 | .21** | .19** | .20** | .31** | .22** | .14** | .03 | .11* | .15** |  |
|  | [-.06, .15] | [-.11, .09] | [.11, .31] | [.09, .28] | [.09, .29] | [.22, .40] | [.12, .31] | [.04, .24] | [-.07, .13] | [.00, .21] | [.05, .25] |  |

*Note*. Values in square brackets indicate the 95% confidence interval for each correlation. ADHD = attention deficit hyperactivity disorder; ALC = alcohol dependence, ANO = anorexia nervosa, ANX = anxiety disorder, AUT = autism spectrum disorder, BPD = bipolar disorder, MDD = major depressive disorder, NEU = neuroticism, OCD = obsessive-compulsive disorder, OPI = opioid dependence, PTSD = posttraumatic stress disorder, SCZ = schizophrenia. For the standardized values *M* = 0, *SD* = 1.

Supplementary Table 3. *Hair cortisol sensitivity analyses.*

|  | Acute Dysfunction vs. Resilient | | | |
| --- | --- | --- | --- | --- |
| Variable | Estimate | CI | *p*-value | odds |
| *Logistic regression main effect* |  |  |  |  |
| Intercept | -2.41 | -3.96;-1.26 | .0003*** | 0.09 |
| Hair cortisol | 0.45 | 0.02; 0.91 | .0450* | 1.56 |
| Distance hair sampling | -0.55 | -1.19; 0.03 | .0738 | 0.57 |
| Sex | 0.80 | -0.45; 2.37 | .2525 | 2.23 |
| Age | -0.58 | -1.12;-0.12 | .0213* | 0.56 |
| GHQ pre lockdown | 0.63 | 0.23;1.06 | .0029** | 1.88 |
|  |  |  |  |  |
| *Logistic regression + hormonal contraceptives* |  |  |  |  |
| Intercept | -2.42 | -3.97;-1.26 | .0003*** | 0.09 |
| Hair cortisol | 0.46 | 0.03; 0.92 | .0424* | 1.58 |
| Distance hair sampling | -0.56 | -1.20; 0.03 | .0742 | 0.57 |
| Sex | 0.85 | -0.44; 2.44 | .2355 | 2.33 |
| Age | -0.60 | -1.14;-0.13 | .0197* | 0.55 |
| GHQ pre lockdown | 0.62 | 0.22; 1.06 | .0031** | 1.87 |
| Hormonal contraceptives | -0.19 | -1.33; 0.87 | .7370 | 0.83 |
|  |  |  |  |  |
| *Logistic regression + hair treatment* |  |  |  |  |
| Intercept | -2.50 | -4.12;-1.30 | .0003** | 0.08 |
| Hair cortisol | 0.59 | 0.13;1.09 | .0156* | 1.80 |
| Distance hair sampling | -0.62 | -1.29;-0.01 | .0546 | 0.54 |
| Sex | 0.42 | -0.95;2.07 | .5737 | 1.52 |
| Age | -0.75 | -1.35;-0.25 | .0067** | 0.47 |
| GHQ pre lockdown | 0.63 | 0.22;1.06 | .0029** | 1.87 |
| Hair treatment | 1.39 | 0.31;2.53 | .0129* | 4.03 |

Supplementary Table 4**.** *Means, standard deviations, and correlations with confidence intervals of the genetic risk factors and hair cortisol concentration.*

| Variable | *M* | *SD* | HCC | g factor | INT-factor | PSY-factor | ND-factor |
| --- | --- | --- | --- | --- | --- | --- | --- |
| HCC | 0.05 | 1.00 |  |  |  |  |  |
|  |  |  |  |  |  |  |  |
|  |  |  |  |  |  |  |  |
| g-factor | 0.00 | 1.00 | .01 |  |  |  |  |
|  |  |  | [-.15, .18] |  |  |  |  |
|  |  |  |  |  |  |  |  |
| INT-factor | 0.00 | 1.00 | .09 | .65** |  |  |  |
|  |  |  | [-.08, .25] | [.59, .71] |  |  |  |
|  |  |  |  |  |  |  |  |
| PSY-factor | -0.00 | 1.00 | -.10 | .42** | -.15** |  |  |
|  |  |  | [-.26, .07] | [.33, .50] | [-.25, -.05] |  |  |
|  |  |  |  |  |  |  |  |
| ND-factor | 0.00 | 1.00 | .01 | .34** | -.17** | -.04 |  |
|  |  |  | [-.16, .17] | [.24, .43] | [-.27, -.07] | [-.14, .06] |  |
|  |  |  |  |  |  |  |  |
| DYS-factor | -0.00 | 1.00 | -.02 | .17** | -.02 | -.04 | -.04 |
|  |  |  | [-.18, .15] | [.07, .27] | [-.13, .08] | [-.14, .06] | [-.14, .06] |
|  |  |  |  |  |  |  |  |

*Note.* M and SD are used to represent mean and standard deviation, respectively. Values in square brackets indicate the 95% confidence interval for each correlation. “HCC” = hair cortisol concentration; “INT” = internalizing disorders; “PSY” = psychotic disorders; “ND” = neurodevelopmental disorders; “DYS” = dysfunctional coping disorders; “g”: general pleiotropic pPRS factor. Bold indicates bivariate logistic regression is significant. * indicates p < .05. ** indicates p < .01.

Supplementary Table 5. *General pleiotropic pPRS factor and interaction with hair cortisol.*

|  | Acute Dysfunction vs. Resilient | | | |
| --- | --- | --- | --- | --- |
| Variable | Estimate | CI | *p*-value | odds |
| *Logistic regression main effect N =364* |  |  |  |  |
| Intercept | -3.66 | -5.10;-2.64 | <.001*** | 0.03 |
| g factor | 0.44 | 0.06;0.83 | .0253* | 1.55 |
| Sex | 1.39 | 0.29;2.85 | .0281* | 4.00 |
| Age | -0.76 | -1.35;-0.27 | .0053** | 0.47 |
| GHQ pre lockdown | 0.52 | 0.15; 0.89 | .0058** | 1.68 |
|  |  |  |  |  |
| *Interaction effect N = 142* |  |  |  |  |
| Intercept | -2.72 | -5.07;-1.20 | .0037** | 0.07 |
| HCC | 0.67 | 0.15;1.28 | .0180* | 1.96 |
| g factor | 0.34 | -0.28;0.99 | .2937 | 1.40 |
| Sex | 0.95 | -0.70;3.27 | .3247 | 2.58 |
| Age | -0.53 | -1.22;0.05 | .0964 | 0.59 |
| GHQ pre lockdown | 0.87 | 0.34;1.48 | .0023** | 2.38 |
| Hair cortisol x g factor | -0.17 | -0.84;0.44 | .5927 | 0.84 |

*Note.* Bivariate logistic regression was calculated considering only genetic risk on the dataset with 364 participants. The genetic and environmental risk interaction was calculated on the complete data set with 142 participants. "g factor" = general pleiotropic pPRS factor. “HCC” = hair cortisol concentration. Sex is coded as males = 0 and females = 1.

Supplementary Table 6. *Factor "neurodevelopmental disorders" and interaction with hair cortisol.*

|  | Acute Dysfunction vs. Resilient | | | |
| --- | --- | --- | --- | --- |
| Variable | Estimate | CI | *p*-value | odds |
| *Logistic regression main effect N =364* |  |  |  |  |
| Intercept | -3.67 | -5.11;-2.65 | <.001*** | 0.03 |
| ND factor | 0.43 | 0.04;0.82 | .0314* | 1.53 |
| Sex | 1.41 | 0.31;2.88 | .0256* | 4.11 |
| Age | -0.72 | -1.29;-0.25 | .0059** | 0.48 |
| GHQ pre lockdown | 0.51 | 0.15;0.88 | .0051** | 1.67 |
|  |  |  |  |  |
| *Interaction N = 142* |  |  |  |  |
| Intercept | -2.84 | -5.28;-1.30 | .0030** | 0.06 |
| HCC | 0.63 | 0.04;1.27 | .0427* | 1.88 |
| ND factor | 0.29 | -0.27;0.88 | .3187 | 1.34 |
| Sex | 1.10 | -0.55;3.52 | .2629 | 3.00 |
| Age | -0.49 | -1.19;0.10 | .1280 | 0.61 |
| GHQ pre lockdown | 0.87 | 0.34;1.49 | .0025** | 2.40 |
| Hair cortisol x g factor | 0.05 | -0.48;0.66 | .8464 | 1.06 |

*Note.* Bivariate logistic regression was calculated considering only genetic risk on the dataset with 364 participants. The genetic and environmental risk interaction was calculated on the complete data set with 142 participants. “ND” = neurodevelopmental disorders; “HCC” = hair cortisol concentration. Sex is coded as males = 0 and females = 1.
